# *Porphyromonas gingivalis* Produce Neutrophil Specific Chemoattractants Including Short Chain Fatty Acids

**DOI:** 10.3389/fcimb.2020.620681

**Published:** 2021-01-19

**Authors:** Agnes Dahlstrand Rudin, Arsham Khamzeh, Vignesh Venkatakrishnan, Tishana Persson, Michael Gabl, Otto Savolainen, Huamei Forsman, Claes Dahlgren, Karin Christenson, Johan Bylund

**Affiliations:** ^1^Department of Oral Microbiology and Immunology, Institute of Odontology, The Sahlgrenska Academy at University of Gothenburg, Gothenburg, Sweden; ^2^Department of Rheumatology and Inflammation Research, Institute of Medicine, The Sahlgrenska Academy at University of Gothenburg, Gothenburg, Sweden; ^3^Chalmers Mass Spectrometry Infrastructure, Department of Biology and Biological Engineering, Chalmers University of Technology, Gothenburg, Sweden

**Keywords:** FFAR2, GPR43, granulocyte, polymorphonuclear leucocyte, periodontitis, acetate, propionate, short chain fatty acid

## Abstract

Neutrophil migration from blood to tissue-residing microbes is governed by a series of chemoattractant gradients of both endogenous and microbial origin. Periodontal disease is characterized by neutrophil accumulation in the gingival pocket, recruited by the subgingival biofilm consisting mainly of gram-negative, anaerobic and proteolytic species such as *Porphyromonas gingivalis*. The fact that neutrophils are the dominating cell type in the gingival pocket suggests that neutrophil-specific chemoattractants are released by subgingival bacteria, but characterization of chemoattractants released by subgingival biofilm species remains incomplete. In the present study we characterized small (< 3 kDa) soluble chemoattractants released by growing *P. gingivalis*, and show that these are selective for neutrophils. Most neutrophil chemoattractant receptors are expressed also by mononuclear phagocytes, the free fatty acid receptor 2 (FFAR2) being an exception. In agreement with the selective neutrophil recruitment, the chemotactic activity found in *P. gingivalis* supernatants was mediated in part by a mixture of short chain fatty acids (SCFAs) that are recognized by FFAR2, and other leukocytes (including monocytes) did not respond to SCFA stimulation. Although SCFAs, produced by bacterial fermentation of dietary fiber in the gut, has previously been shown to utilize FFAR2, our data demonstrate that the pronounced proteolytic metabolism employed by *P. gingivalis* (and likely also other subgingival biofilm bacteria associated with periodontal diseases) may result in the generation of SCFAs that attract neutrophils to the gingival pocket. This finding highlights the interaction between SCFAs and FFAR2 in the context of *P. gingivalis* colonization during periodontal disease, but may also have implications for other inflammatory pathologies involving proteolytic bacteria.

## Introduction

Periodontitis is an inflammatory disease that causes degradation of the alveolar bone and periodontal tissue, with deepened gingival pockets and tooth loss as clinical manifestations (Tonetti et al., 2013). The chronic inflammation that characterizes this disease is initiated and maintained by colonization of oral bacteria along the gingival margin and in the gingival crevices. Periodontitis is not, in its traditional meaning, an infectious disease as tissue invasion of microbes is rare, although it can be seen in particular periodontal conditions (Dahlen et al., 2019). Periodontal disease progression is linked to a compositional change in the oral microbiome, that is characterized by increased amounts of gram-negative, anaerobic and proteolytic bacterial species (Bruno and Loos, 2000). One of these species, *Porphyromonas gingivalis*, is a gram negative, rod-shaped bacterium that is more frequently found in subgingival biofilms from patients with periodontitis, as compared to samples from healthy donors (Griffen et al., 1998). The bacterium is highly proteolytic, equipped with an arsenal of cysteine proteases (gingipains) that can degrade a great variety of host proteins, including immune-regulatory factors and cell adhesion proteins (Hocevar et al., 2018).

Neutrophils constitute an important part of our first line of defense and these professional phagocytes are swiftly recruited from circulation to tissues when reached by signals generated by the presence of microbes and/or tissue damage. Once neutrophils arrive at the inflammatory site, the bacteria should ultimately be killed through phagocytosis or formation of neutrophil extracellular traps (NETs), debris should be cleared and, the resolution phase of the inflammatory reaction should be initiated (Amulic et al., 2012). Accordingly, neutrophils are essential for the host response against bacteria that colonize healthy gingival crevices, as well as periodontal pockets, and interestingly these cells represent the vast majority of leukocytes recruited to the gingival extraepithelial sites (Hajishengallis et al., 2015). Several studies have characterized the leukocyte sub-populations in gingival crevicular fluid (GCF) and have reported that up to 98% of GCF-leukocytes are neutrophils, whereas, e.g., monocytes are typically more or less absent (Attstrom, 1970; Skapski and Lehner, 1976). The mechanisms underlying this neutrophil-specific accumulation in GCF are so far unknown.

All neutrophil chemoattractant receptors are G-protein-coupled receptors (GPCRs), and one critical process triggered by ligation of many GPCRs is a transient increase in intracellular Ca^2+^ concentration, which together with other downstream signaling events leads to directional migration (Dahlgren et al., 2016; Petri and Sanz, 2018). Neutrophils are equipped with a number of different GPCRs that respond to multiple agonists of both endogenous and bacterial origin. Whereas endogenous attractants (e.g., IL-8, LTB_4_) deliver neutrophils from the circulation to tissues, chemotactic factors released by bacteria or damaged host cells (e.g., formylated peptides), or generated on bacterial surfaces (C5a) direct them into close contact with the microbes. Consequently, endogenous chemoattractants play an intermediate role in attracting neutrophils to the vicinity of infection, and bacteria-derived substances are thought to play central roles as end-point chemoattractants capable of guiding the phagocytes all the way to its prey (Heit et al., 2002). While endogenous chemoattractants can direct neutrophils into the gingival tissue in periodontitis, bacteria-derived chemoattractants are needed to explain the migration of neutrophils over the epithelium. However, receptors for the well described bacteria-derived chemoattractants (e.g., the formyl peptide receptors (FPR1 and 2) and the C5a receptor) are expressed by multiple types of leukocytes (Rabiet et al., 2007) and agonists thus generally activate also monocytes (Schiffmann et al., 1975; Marder et al., 1985), the short chain free fatty acid receptor 2 (FFAR2) being an exception as no functional receptor is expressed in monocytes (Bjorkman et al., 2016). The identification of bacteria-derived neutrophil specific chemoattractants, and their corresponding GPCRs, capable of explaining the predominance of neutrophils in GCF is so far lacking.

Short chain fatty acids (SCFAs) are part of a larger group of free fatty acids that are present as intermediate metabolites in circulation and tissues (Rodrigues et al., 2016). Free fatty acids can be subcategorized in short chain fatty acids (C2–C6), medium chain fatty acids (MCFAs; C7–C12) and long chain fatty acids (LCFAs >C12) depending on their carbon chain length. While most MCFAs and LCFAs have dietary origin, a majority of the SCFAs are by-products of carbohydrate fermentation by anaerobic gut bacteria (Alvarez-Curto and Milligan, 2016). SCFAs can also be by-products of *proteolytic* bacterial metabolism and have in fact been demonstrated to form when oral anaerobes (e.g., *P. gingivalis*) utilize proteins as an energy source *in vitro* (Takahashi et al., 2000; Krishnan et al., 2015). SCFAs are sensed by the neutrophil GPCR free fatty acid receptor 2 (FFAR2) (also known as GPR43) (Nilsson et al., 2003; Alvarez-Curto and Milligan, 2016), and a number of pure SCFAs including acetic acid (C2), propanoic acid (C3) and butyric acid (C4) induce *in vitro* chemotaxis of neutrophils *via* this receptor (Le Poul et al., 2003; Maslowski et al., 2009). *In vivo*, fiber digesting gut bacteria have been demonstrated to produce SCFAs that influence neutrophil recruitment in murine models of colitis (Sina et al., 2009). Thus, it would not seem unlikely that neutrophils also sense SCFAs generated by proteolytically active bacteria in GCF using FFAR2.

Factors present in the human subgingival biofilm have previously been reported to induce chemotactic responses in neutrophils *in vitro* (Lindhe and Hellden, 1972) and supernatants from various isolated oral bacterial species can induce directional migration of neutrophils (Van Dyke et al., 1982). Although molecular motifs conserved in all bacterial species, such as formylated peptides known to be neutrophil chemoattractants recognized by the G-protein coupled pattern recognizing FPRs (Schiffmann et al., 1975; Dahlgren et al., 2016), would be likely candidates, the characterization and identification of chemotactic factors generated/secreted by bacteria associated with periodontitis remains incomplete. In this study, we show that small soluble factors released by growing *P. gingivalis* are recognized by neutrophils and induce a rise in the concentration of intracellular Ca^2+^, a response typical for ligation of chemotactic receptors. Accordingly, these factors also induce a chemotactic response in human neutrophils. Interestingly, our data demonstrate a neutrophil selectivity in that neutrophils, but not monocytes are activated by the factors released by *P. gingivalis* as well as by a mixture of the short chain fatty acids with the same composition as that released by the bacteria. Taken together, the data presented suggest that FFAR2 recognition of *P. gingivalis*-derived SCFA may be of particular relevance as pro-inflammatory mediators operating during periodontal disease, and that this neutrophil specific phenomenon could help explain the dominance of neutrophils in GCF.

## Materials and Methods

### Ethics Statement

The study was approved by the regional Ethical board of Gothenburg, Sweden (no. 118-16). Written informed consent was obtained from all individuals who donated peripheral blood. Buffy-coats from healthy donors were obtained from the blood bank at Sahlgrenska University Hospital. Since the buffy coats were provided anonymously and could not be traced back to a specific individual, ethics approval was not needed.

### Isolation of Human Neutrophils From Buffy-Coats and Peripheral Blood

Human neutrophils were isolated from buffy-coats or peripheral blood from healthy blood donors as first described by Boyum (1968). Erythrocytes were removed by dextran sedimentation, followed by centrifugation on Ficoll-Paque to separate granulocytes from mononuclear cells. The remaining erythrocytes were lysed by hypotonic treatment and polymorphonuclear cells were washed three times in Krebs Ringer phosphate buffer (KRG). Neutrophils were resuspended in KRG supplemented with Ca^2+^ (1 mM) and kept on ice until use. For certain experiments, the total leukocyte content of buffy coats was used (separated as above but without Ficoll-Paque).

### Preparation of Bacterial Supernatants

Two laboratory strains (W83 and 381), and two clinical isolates of *P. gingivalis* (P16 (OMGS 711) and P24 (OMGS 766)) obtained from the strain collection at the department of Oral Microbiology University of Gothenburg, Sweden (OMGS) (Dahlen et al., 2007), were used for the preparation of bacterial culture supernatants. Laboratory strains and clinical isolates of *P. gingivalis* were cultured on Brucella agar plates in an anaerobic atmosphere of 80% N_2_, 10% H_2_ and 10% CO_2,_ at 37°C for 48 h. Laboratory strains of *S. aureus* and *S. salivarius* were cultured on blood agar plates in aerobic atmosphere. Colonies were gently removed from plates with an inoculation loop and dissolved in dH_2_O with 10% KRG supplemented with Ca^2+^ (1 mM). For normalization of different supernatants, all bacterial suspensions were diluted to 1 × 10^10^ CFU/ml (based on OD_550 nm_), before centrifugation at 14000×g, for 5 min at 4°C. Supernatants were sterile-filtered through 0.2-μm filters (Corning), freeze-dried and stored at −80°C. Samples of supernatant were cultured to ascertain sterility. Supernatants from agar plates without bacteria, prepared in a similar manner as the bacterial supernatants, were used as negative controls.

Before use, all freeze-dried supernatants were dissolved in dH_2_O (one tenth of the original volume) and centrifuged through size-exclusion filters (Amicon, ultracel 3 kDa, Merck Millipore) for 20 min at 14000×g and 4°C. This step was done to focus on small soluble chemoattractants and to remove LPS and other large bacterial products; endotoxin levels in the final supernatants were < 0.05 EU/ml as determined by limulous amoebocyte lysate test. All experiments shown are from size filtered supernatants prepared in this way.

### Chemotaxis Assay

Isolated neutrophils (2 × 10^6^ cells/ml) from human peripheral blood samples were resuspended in KRG supplemented with Ca^2+^ and 0.3% bovine serum albumin (BSA), and loaded on a 3-μm polycarbonate chemotaxis membrane with hydrophobic mask (ChemoTX Disposable Chemotaxis System; Neuroprobe Inc.). Cells (6 × 10^4^ cells/well) were allowed to migrate over the membrane towards chemotactic stimuli; i.e., bacterial culture supernatants (1:10, 1:20, 1:100 dilutions), SCFAs, fMLF (10 nM) or buffer for 90 min, 37°C, 5% CO_2_. Migrated cells were lysed with 2% cetyltrimethylammonium bromide in phosphate buffered saline (PBS) with 2% BSA, and quantified by measuring myeloperoxidase (MPO) activity in cell lysates with peroxidase reagent (OPD, Sigma) in phosphate citrate buffer (0.05 M, pH 5), according to manufacturer’s instructions. Absorbance at 450 nm was measured in a plate reader (CLARIOstar, BMG Labtech). Values were calculated as a percentage of the positive control (6 × 10^4^ lysed cells). When migration was stimulated with SCFAs, pH was adjusted to be equal in all wells before MPO quantification.

### β-Arrestin Recruitment Assay

The abilities of culture supernatants of the four *P. gingivalis* strains to activate formyl peptide receptors 1 and 2 were evaluated using PathHunter β-arrestin GPCR cell lines (DiscoverX) that express FPR1 and FPR2, respectively (Gabl et al., 2017). The PathHunter β-arrestin recruitment assay is based on enzyme fragment complementation technology; CHO-K1 cells express GPCRs tagged with enzyme an fragment (PK) and β-arrestin tagged with the corresponding enzyme fragment (EA). Activation of the receptor induces the recruitment of β-arrestin, facilitating complementation of the two enzyme fragments. The functional enzyme hydrolyses a substrate, generating a chemiluminescent signal that was measured in a plate reader (CLARIOStar).

### Measurement of Neutrophil Cytosolic Ca^2+^ Concentration by Flow Cytometry

Isolated neutrophils, or total leukocytes from human buffy-coats were pelleted and resuspended in cell loading medium (KRG supplemented with Ca^2+^ with 1% heat-inactivated FCS) at 1 × 10^7^ cells/ml. Cells were loaded with Fluo-3 AM (3.6 µl/ml) and Fura-Red (10 µl/ml) and incubated for 30 min at 37°C, washed twice and resuspended in cell-loading medium at 6 × 10^6^ cells/ml. Before analysis, cells were incubated for 5 min at 37°C (with/without GPCR receptor antagonist (**Table 1**) or allosteric modulator (Cmp58; Calbiochem, 1 µM). Baseline fluorescence was monitored for 20 to 30 s and stimulus was added with a pipette fitted with a gel-tip. Fluorescence emission was analyzed simultaneously in FL-1 and FL-3 and a ratio between the curves was calculated (Novak and Rabinovitch, 1994).

**Table 1:** Agonists and antagonists, directed towards G-protein coupled receptors (GPCRs), used to stimulate and inhibit neutrophil intracellular Ca^2+^ signals.

GPCR	Receptor agonist	Final Conc. (nM)	Receptor antagonist	Final Conc. (μM)
FPR1	fMLF (Sigma)	1	Cyklosporin H (Abcam)	1
FPR2	WKYMVM (Alta Bioscience)	10	PBP10 (Calbiochem, Merck)	1
CXCR2	Gro-α (R&D)	12.5	SB225002 (Calbiochem, Merck)	10
PAFR	PAF (Avanti, Sigma)	10	WEB (Tocris)	1
GPR43	Acetate (Merck)	10^6^	CATPB/GLPG0974 (Sigma)/(Tocris)	1
P2Y2R	ATPγS (Sigma)	5000	AR-C 118925XX (Tocris)	1
C5aR	C5a (R&D)	6	W54011 (Merck)	1
GPR84	Zq16 (Tocris Bioscience)	100	GLPG1205 (Galapagos NV)	0.5

FPR1, formyl peptide receptor 1; FPR2, formyl peptide receptor 2; PAFR, platelet activating factor receptor; fMLF, formyl-Met-Leu-Phe; PAF, platelet activating factor.

When the total leukocyte population was used, cells were, after labeling with calcium dyes, also stained with anti-CD45 antibody (Abcam, diluted 1:500) for 20 min, at 4°C. CD45 is differentially expressed on distinct leukocyte subsets and facilitate the gating on neutrophils, monocytes and lymphocytes. The cell types (neutrophils, monocytes and lymphocytes) were gated based on CD45 and side scatter properties and the Ca^2+^ responses of the different cell types were analyzed separately (**Figure 6B**), as previously described (Welin et al., 2015).

### Solid Phase Extraction

To separate hydrophobic and hydrophilic molecules, the size sorted (< 3 kDa) bacterial supernatants were passed through a C18 solid phase extraction (SPE) cartridge (Strata C18-E, pore size 73 Å, Phenomenex), according to manufacturer’s instructions. Unbound flow through, containing the hydrophilic fraction, was collected and stored at -20°C. Retained molecules were eluted from the C18 column using 40% (V/V) acetonitrile containing 0.05% (V/V) formic acid, dried and resuspended in KRG supplemented with Ca^2+^ with 1% DMSO (Sigma).

### Liquid Chromatography and Tandem Mass Spectrometric Analysis

Sample derivatization: 10 µl of *P. gingivalis* culture supernatant, 10 μl methanol (75%), 10 μl 200 mM 3-NPH (3-nitrophenylhydrazine in MeOH (75%)), 10 μl 120 mM EDC-6% pyridine (N-(3-Dimethylaminopropyl)-N-ethylcarbidiimide in 75% MeOH with 6% pyridine) was mixed and incubated 45 min, RT with shaking. The derivatization reaction was quenched with 10 μl of 200 mM quinic acid in MeOH, mixed and incubated 15 min, RT with shaking. 950 μl of water was added to the samples followed by centrifugation at 15,000×*g*, RT, for 5 min. 100 μl of supernatant and 100 μl of internal standard (13-C 3-nitrophenylhydrazine labeled short chain fatty acids) transferred to HPLC vials. Samples were analyzed immediately or kept at 4°C until analysis.

The samples were analyzed with LC-MS/MS consisting of an ExionLC UHPLC system coupled to a 6500+ QTRAP (both from AB Sciex LLC, Framingham, USA). The analytes were separated in a Phenomenex Kinetex C18 (100 × 2.1 mm, 1.7 μm, 100 Å) column, at 40°C, following gradient: 0–3 min 0.5% B, 3.00–3.01 min 0.5–2.5% B, 3.01–6.00 min 2.5–17% B, 6.00–10.00 min 17–45% B and 10.00–13.00 min 45–55% B, followed by washing and re-equilibration of the column. Mobile phase A and B were water and acetonitrile, respectively, and total flow was set to 0.4 mL/min. APCI ionization was used in positive polarity and the analytes were detected using optimized MRM-transitions for each analyte and internal standard. A calibration curve covering the range of the analytes in the samples was injected together with the analytes.

### Statistical Analyses

Non-parametric statistical tests were used to determine significance. All statistical analyses were performed in GraphPad Prism software (version 8.2.1; San Diego, CA, USA). A *p*-value less than 0.05 was considered statistically significant and level of significance is indicated in the figures: ns >0.05, *< 0.05, **< 0.01, ***< 0.001. The statistical tests used are specified in the figure legends.

### Funding Sources

The work was supported by the Swedish Research Council (HF, 02448; JB, 2019-01123), the Swedish Foundation for Strategic Research (HF, SM-17-0046), and the Swedish state under the ALF-agreement (CD, ALFGBG 72510; HF, ALFGBG 78150) and TUA-agreement (JB, 917531). The sponsors did not have any role in any part of the study.

## Results

### Supernatants From *P. gingivalis* Cultures Induce Neutrophil Chemotaxis and Trigger Intracellular Ca^2+^ Signals

**Figure 1:**
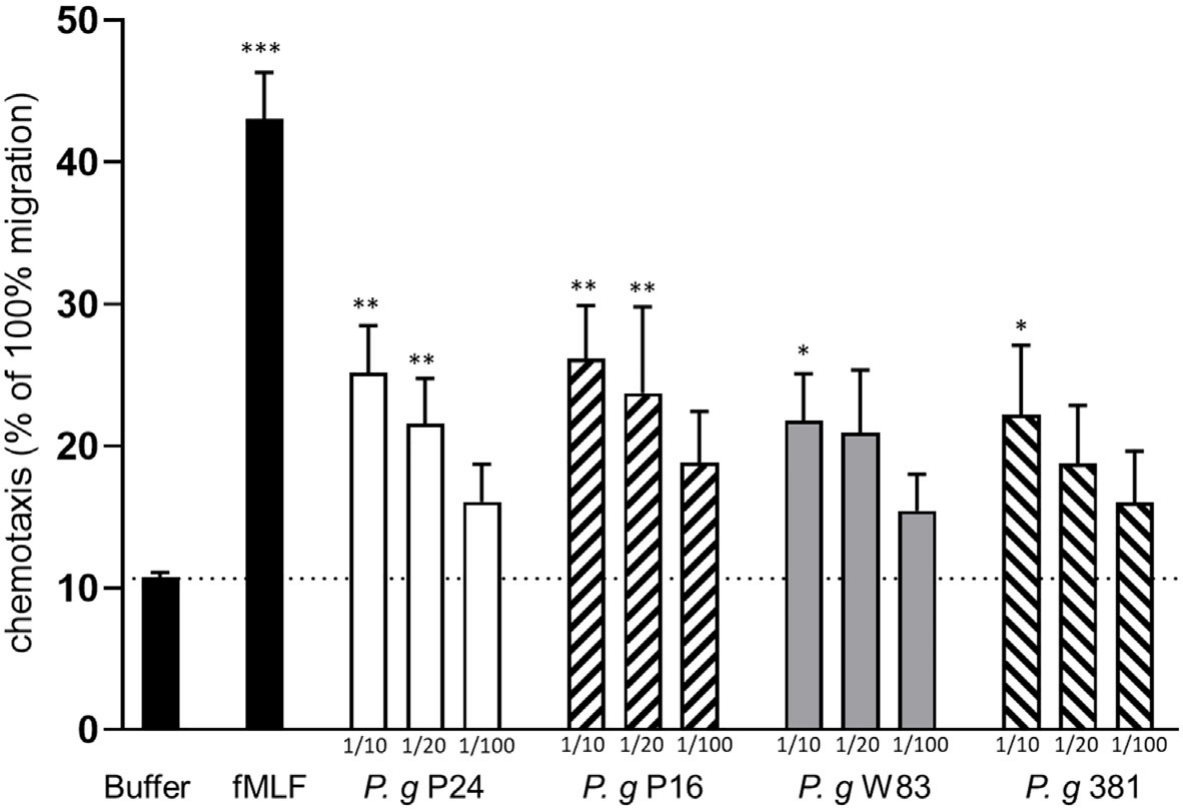
Culture supernatants of *P. gingivalis* induce chemotaxis of human neutrophils. Neutrophils isolated from peripheral blood of healthy humans were allowed to migrate over a ChemoTx membrane towards chemotactic stimuli (bacterial supernatants (1:10, 1:20, 1:100), fMLF (10 nM)) or buffer) for 90 min. Migrated cells were quantified by measurement of myeloperoxidase activity after cell lysis. Six independent experiments were performed and each stimulus was analyzed in triplicates (technical repeats). Values shown are calculated as percentage of positive control (100% migration of cells) and are presented as mean values with SEM. Friedman test followed by Dunn´s multiple comparison test was used for statistical analysis. *< 0.05, **< 0.01, ***< 0.001.

We investigated whether small (<3 kD) soluble factors derived from two established lab strains (W83 and 381), and two clinical isolates (P16 and P24) of *P. gingivalis* were able to induce neutrophil chemotaxis *in vitro*. Supernatants from bacterial cultures, in three different dilutions (1:10, 1:20 and 1:100), were used as chemotactic stimuli and the potent neutrophil chemoattractant fMLF was used as a positive control. Supernatants prepared from cultures of all four strains induced neutrophil chemotaxis in a dose-dependent manner (**Figure 1**).

**Figure 2:**
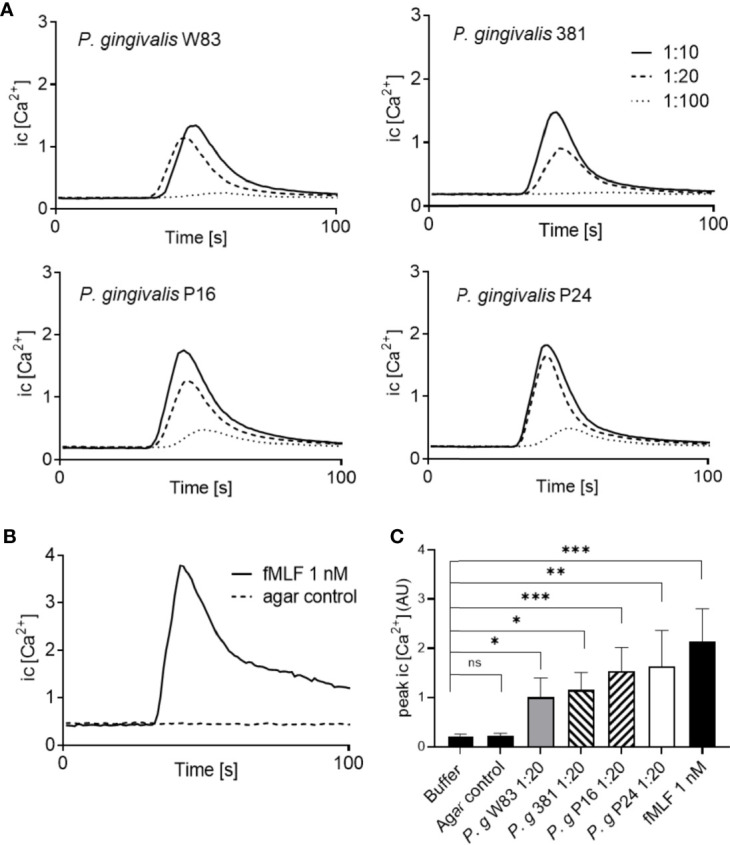
*P. gingivalis* supernatants trigger transient Ca^2+^ signals in neutrophils. Neutrophils isolated from buffy-coats from healthy blood donors were co-stained with Ca^2+^ dyes Fura-Red and Fluo-3. The fluorescence emission, after stimulation, was analyzed simultaneously in FL-1 and FL-3 and the curves represent a ratio between Fluo-3 and Fura-Red fluorescence. **(A)** Neutrophil stimulation with *P. gingivalis* supernatants induced transient rises in intracellular Ca^2+^ concentration in a dose-dependent manner. **(B)** A rise in intracellular Ca^2+^ concentration was also induced by fMLF (1 nM), while no changes in intracellular Ca^2+^ concentration were seen after stimulation with agar control supernatants (without bacteria). **(C)** Peak values of neutrophil intracellular Ca^2+^ signals after stimulation with the four *P. gingivalis* culture supernatants (1:20) (mean + SD, n = 5–13). Kruskal-Wallis test followed by Dunn´s multiple comparison test was used for statistical analysis. ns >0.05, *<0.05, **< 0.01, ***< 0.001.

Ligand binding to neutrophil chemoattractant GPCRs typically induces a transient rise of free Ca^2+^ in the cytoplasm (Metzemaekers et al., 2020). All four *P. gingivalis* supernatants gave rise to dose dependent intracellular Ca^2+^ signals in human neutrophils (**Figures 2A, C**), comparable to the rapid, transient Ca^2+^ signal induced by fMLF (**Figures 2B, C**) and with significantly higher peak values as compared to buffer control (**Figure 2C**). Importantly, no Ca^2+^ signals were detected after stimulation of neutrophils with supernatants from empty agar plates without bacteria (**Figures 2B, C**). Collectively, these data demonstrate that *P. gingivalis* indeed releases small, soluble chemoattractants recognized by human neutrophils.

Aiming to identify specific chemoattractants in supernatants by the use of specific receptor agonists and antagonists turned out fruitless; antagonists for a number of different neutrophil GPCRs were unable to block the Ca^2+^ signals induced by *P. gingivalis* supernatants (**Table 1** and **Supplementary Figures 1** **and** **2**). This is likely due to the fact that the supernatants contain a complex mixture of biomolecules and the response is thus the result of several distinct receptors activated simultaneously.

### *P. gingivalis* Supernatants Contain Very Little or No Formylated Peptides

**Figure 3:**
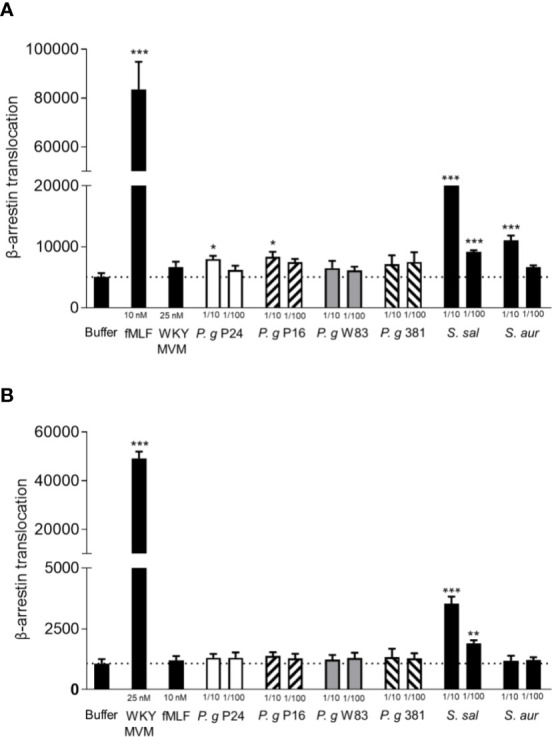
*P. gingivalis* supernatants of do not activate FPR1 or FPR2. Activation of FPR1 and FPR2 was measured as the ability of bacterial culture supernatants to stimulate recruitment of β-arrestin in CHO cells overexpressing FPR1 and FPR2 respectively, and β-arrestin recruitment was quantified using an enzyme fragment complementation assay. Two experiments were performed with the same cell line, and each condition was analyzed in triplicates. **(A)** Culture supernatants of the two *P. gingivalis* lab strains (W83, 381) (1:10, 1:100) did not stimulate β-arrestin recruitment in cells expressing FPR1, while supernatants of the clinical isolates of *P. gingivalis* (P24, P16) showed slight FPR1 activity in the higher concentration (1:10). The FPR1 agonist fMLF (10 nM) and control supernatants of *S. salivarius* and *S. aureus* did stimulate β-arrestin recruitment (mean + SD). **(B)** Culture supernatants of the four *P. gingivalis* strains (1:10, 1:100) and *S. aureus* (1:10, 1:100) did not stimulate β-arrestin recruitment in cells expressing FPR2, while FPR2 agonist WKYMVM (25 nM) and control supernatant of *S. salivarius* (1:10, 1:100) did stimulate β-arrestin recruitment (mean + SD). Statistics were calculated as 6 technical repeats and Kruskal-Wallis test followed by Dunn´s multiple comparison test was used to analyze significant differences. *<0.05, **< 0.01, ***< 0.001.

Since formylated peptides are well documented chemoattractants, recognized by neutrophils using one or both of its two formyl peptide receptors (FPR1 and 2) and released by a number of different bacteria (Marasco et al., 1984; Rot et al., 1986; Southgate et al., 2008), we tested whether the supernatants contained any FPR ligands in an FPR-mediated arrestin translocation system. Reporter cell lines (overexpressing either FPR1 or FPR2 and providing a signal when the activated receptor bind to β-arrestin) that are highly sensitive to detect β-arrestin recruiting FPR ligands (Forsman et al., 2011; Forsman et al., 2012a; Gabl et al., 2017) were stimulated with *P. gingivalis* supernatants. The two lab strains (W83 and 381) induced no measurable FPR1 activation, and the two clinical isolates (P24 and P16) induced very low levels of FPR1 activation (**Figure 3A**). Control supernatants from two unrelated bacteria, *Streptococcus salivarius* and *Staphylococcus aureus* did activate FPR1 in a dose-dependent manner (**Figure 3A**). This indicates that the levels of FPR1 agonists with ability to recruit β-arrestin present in *P. gingivalis* supernatants are very low or undetectable.

Formyl peptide receptor 2 (FPR2) is a related receptor to FPR1 and structurally very similar, sharing 69% of protein sequence (He et al., 2014). While fMLF is a weak agonist for FPR2, other (longer) formylated peptides are potent agonists (Kretschmer et al., 2010; Forsman et al., 2012b). In contrast to the *S. salivarius* supernatant, neither of the *P. gingivalis* supernatants induced measurable FPR2 activation (**Figure 3B**). These results indicate that *P. gingivalis* release no or very little formylated peptides. In support of this conclusion, the *P. gingivalis* supernatants were incapable of activating the neutrophil NADPH-oxidase to release reactive oxygen species (ROS) (**Supplementary Figure 3**), which is one major effect of FPR-activation apart from chemotaxis (Dahlgren et al., 2016).

### Only the Hydrophilic Fraction of the *P. gingivalis* 381 Supernatant Contain Factors With Neutrophil Activating Capacity

**Figure 4:**
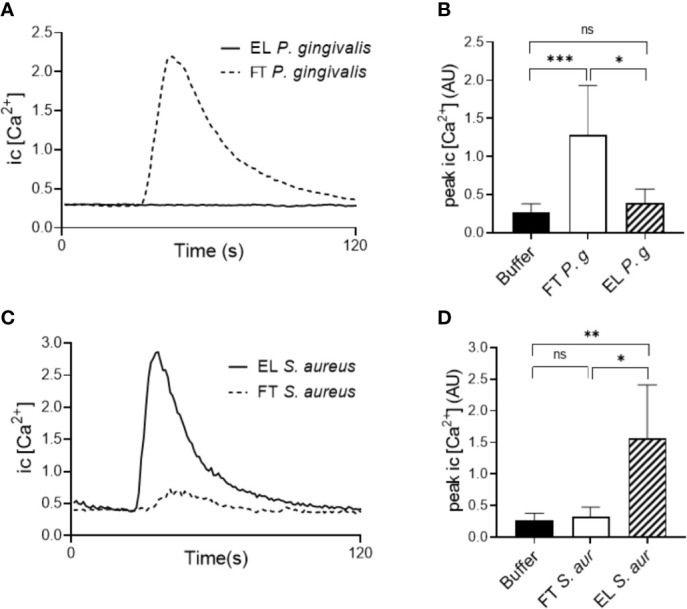
Biochemical separation of bacterial supernatants. The components of supernatants of *P. gingivalis* 381 and *S. aureus* were purified by C18 cartridge sorting to separate hydrophobic (Eluate (EL)) and hydrophilic (Flow through (FT)) molecules. Human buffy-coat neutrophils were stimulated with the EL and FT fractions of either culture supernatants and intracellular Ca^2+^ concentration was measured by flow cytometry. **(A)** Graph shows the neutrophil intracellular Ca^2+^ concentration after stimulation with the *P. gingivalis* 381 EL- and FT fractions from one representative experiment. **(B)** Peak values of neutrophil Ca^2+^ signals after *P. gingivalis* 381 EL- and FT fraction stimulation (mean + SD, n = 8). **(C)** Graph shows the neutrophil intracellular Ca^2+^ concentration after stimulation with the *S. aureus* EL- and FT fractions from one representative experiment. **(D)** Peak values of neutrophil Ca^2+^ signals after *S. aureus* EL- and FT fraction stimulation (mean + SD, n = 6). Kruskal-Wallis test followed by Dunn´s multiple comparison test was used for statistical analysis. ns >0.05, *<0.05, **< 0.01, ***< 0.001.

We next utilized a biochemical approach to separate the complex *P. gingivalis* (strain 381) supernatant based on hydrophobicity using C18 reversed phase SPE cartridges. Whereas hydrophilic molecules pass through the C18 cartridge and are collected as the flow through fraction (FT), hydrophobic components are retained and later eluted (EL fraction) using an organic solvent. The two fractions of the *P. gingivalis* supernatant (FT and EL) were then used to stimulate intracellular Ca^2+^ signaling and chemotaxis in neutrophils. All activity, both Ca^2+^ signaling (**Figures 4A, B**) and chemotaxis (**Supplementary Figure 4**), was found in the FT fraction of the *P. gingivalis* supernatant and no activity was found in the EL fractions. This was in sharp contrast to supernatants from *S. aureus* (**Figures 4C, D**) and *S. salivarius* (not shown), where all Ca^2+^ signaling activity was recovered in the EL fraction. This fits well with the absence of FPR activation in *P. gingivalis* supernatants (**Figure 3**) and indicate that the chemoattractants from *P. gingivalis*, but not from *S. aureus* or *S. salivarius*, are hydrophilic.

### *P. gingivalis* Supernatants Contain SCFAs

The identification of small, hydrophilic molecules from *P. gingivalis* supernatants as capable of triggering neutrophil activation would fit well with the presence of SCFAs as they are described end-products of proteolytic metabolism and they are highly hydrophilic molecules known to ligate the neutrophil GPCR FFAR2 (Bjorkman et al., 2016; Rodrigues et al., 2016). In contrast to formylated peptides that are extremely potent chemoattractants (nanomolar doses fMLF are enough to attract neutrophils *in vitro* (e.g., **Figure 1**)), SCFAs reportedly require high micromolar (or even millimolar) levels in order to activate cells *in vitro* (Le Poul et al., 2003; Sina et al., 2009). To detect and quantify SCFAs from *P. gingivalis*, we performed liquid chromatography and tandem mass spectrometric analyses on the supernatants. The presence of low to medium-high micromolar concentrations of acetic acid, propanoic acid, butyric acid, iso-butyric acid, succinic acid, iso-valeric acid and caproic acid were found in the supernatants of all four *P. gingivalis* strains (**Table 2**). Valeric acid was not detectable in either of the supernatants tested.

**Table 2:** Concentration (µM) of SCFAs in culture supernatants of *P. gingivalis* strains.

Supernatant	Acetic acid	Propanoic acid	Butyric acid	Iso-butyric acid	Succinic acid	Valeric acid	Iso-valeric acid	Caproic acid
*P. gingivalis* W83	29.8	10.7	35.4	6.6	21.1	ND	14.1	0.6
*P. gingivalis* 381	105.0	19.6	85.0	16.8	17.5	ND	32.7	0.6
*P. gingivalis* P16	108.1	27.5	97.4	11.4	24.5	ND	26.6	0.2
*P. gingivalis* P24	32.2	7.5	24.9	3.5	22.5	ND	8.2	0.1

**Figure 5:**
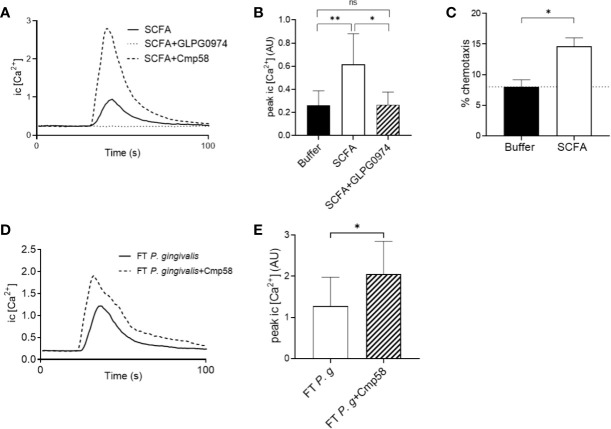
Neutrophil cytosolic Ca^2+^ signaling and chemotaxis in response to SCFAs found in *P. gingivalis* supernatants **(A)** A mixture of SCFAs, prepared based on the concentrations of SCFAs found in the supernatants of *P. gingivalis*, was used to stimulate neutrophils in the presence or absence of an FFAR2 antagonist (GLPG0974) or the allosteric modulator (Cmp58). Intracellular Ca^2+^ responses were monitored by flow cytometry. Graph shows the neutrophil intracellular Ca^2+^ response after stimulation with the SCFAs ± GLPG0974 or Cmp58 from one representative experiment. **(B)** Peak values of neutrophil intracellular Ca^2+^ signals after SCFA stimulation in the presence or absence of GLPG0974 (mean + SD, n = 9). Kruskal-Wallis test followed by Dunn´s multiple comparison test was used to calculate significant differences. **(C)** Neutrophils isolated from peripheral blood of healthy donors were allowed to migrate over a ChemoTx membrane towards chemotactic stimuli (i.e., SCFA or buffer) for 90 min (n=7). Migrated cells were quantified by measurement of myeloperoxidase activity after cell lysis. Values shown are calculated as percentage of positive control (100% migration) and are presented as mean values with SEM. Wilcoxon matched-pairs signed rank test was used to analyze statistics. **(D)** Human buffy-coat neutrophils were stimulated with the FT fraction of *P. gingivalis* 381 supernatant (± Cmp58), and intracellular Ca^2+^ responses were followed by flow cytometry. Graph shows the neutrophil intracellular Ca^2+^ concentration after stimulation with the *P. gingivalis* 381 FT fraction in the presence or absence of the allosteric modulator (Cmp58) from one representative experiment. **(E)** Peak values of neutrophil intracellular Ca^2+^ signals after *P. gingivalis* FT fraction stimulation with or without addition of Cmp58 (mean + SD, n = 8). Wilcoxon matched-pairs signed rank test was used to analyze statistics. ns >0.05, *<0.05, **< 0.01.

A mixture of the SCFAs (acetic acid 100 µM, propanoic acid 25 µM, butyric acid 100 µM, iso-butyric acid 10 µM, succinic acid 25 µM, iso-Valeric acid 25 µM, caproic acid 0.2 µM) with the same composition and at concentrations similar to those found in the culture supernatants of *P. gingivalis* was prepared and found to trigger significant neutrophil Ca^2+^ signaling (**Figures 5A, B**) as well as chemotaxis (**Figure 5C**). As expected, the effect of the SCFA mix was solely mediated through FFAR2, since the induced Ca^2+^ response was completely blocked by the FFAR2 antagonist GLPG0974 (**Figures 5A, B**). In addition, FFAR2 activity is known to be potently enhanced by the presence of the allosteric modulator Cmp58 (Martensson et al., 2018). Ca^2+^ responses to the SCFA mix were greatly enhanced by Cmp58 (**Figure 5A**).

The allosteric FFAR2 modulator Cmp58 significantly enhanced also the Ca^2+^ signals induced by the hydrophilic fraction (FT) from *P. gingivalis* supernatants (**Figures 5D, E**), indicating that FFAR2 ligation makes up at least part of this response. However, the FT-triggered Ca^2+^ signals could not be completely abrogated with either of the FFAR2 antagonists (GLPG0974 or CATPB) (**Supplementary Figure 5**).

### SCFAs Released by *P. gingivalis* as Neutrophil Specific Chemoattractants

**Figure 6:**
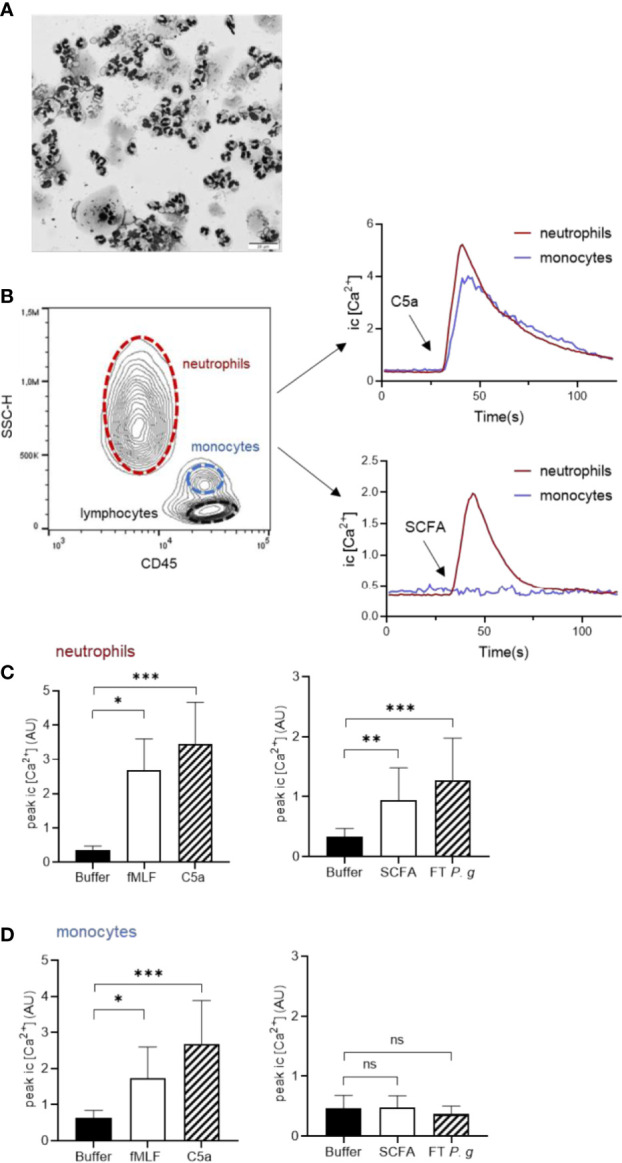
SCFAs released by *P. gingivalis* activate neutrophils, but not monocytes. **(A)** GCF samples from patients diagnosed with periodontitis were cytospun and stained with Giemsa and May-Grünwald and were evaluated microscopically as described earlier (Dahlstrand Rudin et al., 2020). Micrograph shows abundant neutrophils, a few epithelial cells and complete absence of mononuclear cells. The scale bar represents 20 µm. **(B)** Human buffy-coat leukocytes, costained with Ca^2+^ dyes Fluo-3 and Fura Red and anti-CD45 antibody, were stimulated with fMLF (1 nM), C5a (100 ng/ml), SCFA, FT fraction of *P. gingivalis* 381 supernatant, or buffer and intracellular Ca^2+^ signals were monitored by flow-cytometry. Neutrophils and monocytes were gated based on CD45 and side-scatter properties and analyzed separately. Graphs show the neutrophil and monocyte intracellular Ca^2+^ responses after stimulation with C5a, and SCFA from one representative experiment. Graphs show peak values (mean + SD, n = 5-9) of Ca^2+^ responses for neutrophils **(C)** and monocytes **(D)**, respectively after stimulation with fMLF, C5a, SCFAs, FT *P. g* or buffer. Kruskal-Wallis test followed by Dunn´s multiple comparison test was used for statistical analyses ns >0.05, *<0.05, **< 0.01, ***< 0.001.

Having established that *P. gingivalis* cultured *in vitro* is capable of producing high levels of different SCFAs that can be recognized by FFAR2 to drive neutrophil chemotaxis, we next wanted to test whether this ligand-receptor interaction is specific for neutrophils and could help explain why neutrophils, out of all leukocytes, is so dominating in the GCF of periodontitis patients. It is well established that neutrophils are by far the most abundant leukocyte in GCF (Silva et al., 2019) and in freshly drawn GCF samples from periodontitis patients, neutrophils were found in abundance, whereas, e.g., monocytes were notably absent (**Figure 6A**) (Dahlstrand Rudin et al., 2020). This selective recruitment of neutrophils (and not other leukocytes) indicates that neutrophil specific chemoattractants are operating at this inflamed site. The classic chemoattractants often discussed in the context of neutrophil infiltration to infected or colonized sites are formylated peptides and the anaphylatoxin C5a (formed by complement activation on the bacterial surface) (Heit et al., 2002).

We stimulated mixed leukocyte samples with fMLF, C5a, SCFAs, or *P. gingivalis* FT fraction in a flow cytometric system capable of monitoring cell-type specific responses (Welin et al., 2015). By gating the different cell types (neutrophils, monocytes and lymphocytes) based on side scatter and CD45 properties, we were able to monitor Ca^2+^ responses of these leukocyte subsets simultaneously (**Figure 6B**). Whereas fMLF and C5a triggered calcium signals in both neutrophils and monocytes, the SCFA mix and FT fraction of *P. gingivalis* only induced calcium responses in neutrophils (**Figures 6C, D**). Neither of the stimuli (fMLF, C5a, SCFA mix, or FT) induced intracellular Ca^2+^ signaling in lymphocytes (data not shown).

## Discussion

The capacity of bacteria in subgingival biofilms to induce neutrophil chemotaxis have been established previously (Lindhe and Hellden, 1972), however the chemoattractants released by oral microbes were yet to be identified. We found that the supernatants of the four strains of *P. gingivalis* (P16, P24, W83, 381) contained soluble bacterial products that stimulate neutrophil chemotaxis *in vitro*. One of our previous studies showed that neutrophil intracellular Ca^2+^ signals can be triggered by stimulation with a supernatant derived from a cultured periodontitis bacterial sample, which along with directional migration, is a characteristic neutrophil response to ligation of chemotactic GPCRs (Dahlstrand Rudin et al., 2020). In line with this, the culture supernatants of all four *P. gingivalis* strains triggered a transient increase in neutrophil intracellular Ca^2+^ concentration. The *P. gingivalis* induced intracellular Ca^2+^ flux was, however, not blocked by antagonists directed against an array of well-known neutrophil GPCRs, including the SCFA, receptor FFAR2. The fact that the FFAR2 antagonist was unable to block Ca^2+^ signaling triggered by *P. gingivalis* supernatants, despite the presence of high concentrations of SCFAs, is likely attributed to a complex composition of the *P. gingivalis* supernatants. This means that even though SCFAs contribute to the chemotactic activity, these compounds are by no means the only molecules in the supernatants capable of neutrophil activation.

Prokaryotic and mitochondrial protein synthesis is initiated with formyl methionine, which is removed post-translation. Formylated peptides can, therefore, theoretically be derived from the terminal regions of newly synthesized proteins, released in infected tissues when the bacterial cell wall is disrupted or when mitochondria are lysed at sites of tissue damage (Carp, 1982; Laursen et al., 2005). Several bacterial species including *Escherichia coli*, *Staphylococcus aureus* and *Listeria monocytogenes*, have been shown to release formylated peptides (Marasco et al., 1984; Rot et al., 1986; Southgate et al., 2008), and these formylated peptides are potent FPR1 agonists (Durr et al., 2006). In contrast, our results show that *P. gingivalis* supernatants, surprisingly, contained very low or undetectable levels of FPR agonists able to provoke responses in the β-arrestin assay. One could speculate that *P. gingivalis* is so proteolytic that even small formylated peptides are degraded extracellularly by their impressive arsenal of proteolytic enzymes.

Since we were unable to identify a sole receptor as responsible for the neutrophil activation triggered by *P. gingivalis* supernatants, we decided to separate the complex material based on hydrophobicity. Reversed phase SPE revealed that the activating factors present in the *P. gingivalis* culture supernatant are agonists with hydrophilic properties. In stark contrast, the control culture supernatant of *S. aureus* contained mainly hydrophobic agonists. This suggests that the soluble GPCR agonists released by *P. gingivalis* and *S. aureus* respectively are profoundly different, and further support our results indicating that the *P. gingivalis* chemoattractants are not formylated peptides as these typically contain nonpolar residues (Leu, Phe) (Schiffmann et al., 1975), and would likely be retained by the C18 cartridge. Earlier studies have shown that *S. aureus* releases formylated peptides into culture supernatants (Rot et al., 1986; Southgate et al., 2008), and they are probably active factors in the EL fraction from *S. aureus* supernatants.

Supernatants from *P. gingivalis* contained micromolar concentrations of acetic acid (29.8–108.1 µM), propanoic acid (7.5–27.5 µM), butyric acid (24.9–97.4 µM), iso-butyric acid (3.5–16.8 µM), succinic acid (17.5–24.5 µM) and iso-valeric acid (8.2–32.7 µM). There are earlier reports on millimolar concentrations of acetic acid, butyric acid, propionic acid, iso-butyric acid and iso-valeric acid in culture supernatants of *P. gingivalis* (Takahashi et al., 2000; Diaz and Rogers, 2004). However, as neither growth conditions, method of measurement nor bacterial density in the previous studies were identical to or ours, we were not surprised to see discrepancies in SCFA end concentrations. Moreover, the SCFA concentration at the *in vivo* periodontal lesion is clearly not a fixed parameter. Varying concentrations of SCFAs have also been detected in GCF depending on periodontal health status and gingival inflammation (Niederman et al., 1996; Niederman et al., 1997). Niederman et al. found that the concentration of propanoic and butyric acid in GCF were below detection limits in periodontally healthy gingival sites, present in µM concentrations in GCF from sites diagnosed with mild periodontitis and that they reached mM concentrations in GCF from sites with severe periodontitis (Niederman et al., 1997). SCFA concentration in GCF was also positively correlated to increasing bacterial load of a number of anaerobic bacterial species (Niederman et al., 1997). Consequently, the *in vivo* concentrations of SCFAs in GCF are highly variable, and seem to increase with periodontal disease severity and bacterial load.

The mixture of SCFAs, with similar concentrations of the individual fatty acids as those detected in the *P. gingivalis* supernatants, induced both Ca^2+^ signaling and chemotaxis in human neutrophils *in vitro*. Pre-treatment of cells with FFAR2 antagonist (GLPG0974) or an allosteric FFAR2 modulator respectively confirmed that the SCFA triggered Ca^2+^ signals were indeed mediated *via* FFAR2. This is in coherence with earlier studies showing FFAR2 dependent Ca^2+^ signaling after stimulation with the individual SCFAs; acetic acid, propanoic acid, and butyric acid (Nilsson et al., 2003; Maslowski et al., 2009). Evidence of individual SCFAs acting as neutrophil chemoattractants *in vitro* have been presented before and it seems clear that they are recognized by neutrophils using FFAR2 (Le Poul et al., 2003; Maslowski et al., 2009; Sina et al., 2009; Rodrigues et al., 2016). However, the effect of FFAR2 activation on neutrophil function seems to vary depending on the nature of the agonist; Björkman et al. found no effect on neutrophil chemotaxis after stimulation with the synthetic FFAR2 agonist Cmp1 (Bjorkman et al., 2016), while another synthetic FFAR2 agonist (Phenylacetamide 1) proved to be a potent inducer of neutrophil chemotaxis (Maslowski et al., 2009). The Ca^2+^ signals induced by the FT fraction of the *P. gingivalis* supernatant were clearly amplified in the presence of Cmp58, confirming FFAR2 involvement in signal transduction. However, the fact that FT-triggered Ca^2+^ signals could not be completely abrogated with either of the FFAR2 antagonists (GLPG0974 or CATPB) (**Supplementary Figure 4**) shows that SCFAs are not the only active hydrophilic chemoattractants produced by *P. gingivalis*.

It is well established that neutrophils are the most abundant leukocytes in GCF (80–98% of leukocytes) (Attstrom, 1970; Skapski and Lehner, 1976; Wilton et al., 1976; Kowolik and Raeburn, 1980; Delima and Van Dyke, 2000), which could be the result of leukocyte recruitment being governed by chemoattractants that specifically activate these cells. Formylated peptides and C5a are classic chemoattractants capable of guiding neutrophils to bacteria (Kim and Haynes, 2012). However, neither formylated peptides nor C5a can be considered to be neutrophil specific as their receptors (FPR1 and C5aR, respectively) are expressed in both neutrophils and monocytes, and stimulate directional migration in both cell types (Schiffmann et al., 1975; Marder et al., 1985). Although monocytes have been reported to express FFAR2 on the mRNA level (Senga et al., 2003), the receptor seems to be functionally expressed exclusively in neutrophils (Bjorkman et al., 2016). This is perfectly in line with our data showing that whereas both monocytes and neutrophils respond to fMLF or C5a stimulation, only neutrophils respond to the SCFA mix. The same was true for the hydrophilic fraction (FT) of *P. gingivalis* supernatants. The neutrophil-specific expression of (functional) FFAR2 indicate that SCFAs released by *P. gingivalis* could be one important factor that contribute to neutrophil specific chemotaxis into the gingival pocket.

In the present study, we show that *P. gingivalis* release a mixture of SCFAs that induces FFAR2 mediated intracellular Ca^2+^ signals and chemotaxis in human neutrophils. The functional effects of SCFAs also proved to be neutrophil specific, which further strengthen their role as important chemoattractants in the interplay between periodontal microbes and host defense. Furthermore, as the proteolytic metabolism generating SCFAs is a common trait of the anaerobic bacterial species that colonize the deep periodontal pocket, our finding could also apply to other oral anaerobes as well as other inflammatory pathologies driven by colonization/infection by proteolytic bacteria.

## Data Availability Statement

The raw data supporting the conclusions of this article will be made available by the authors, without undue reservation.

## Author Contributions

ADR, OS, HF, CD, KC, and JB participated in research design. Experiments were conducted by ADR, AK, TP, and OS. Results were analyzed by ADR, AK, VV, TP, MG, KC, and JB. ADR and JB wrote the paper, which was critically revised by all authors. All authors contributed to the article and approved the submitted version.

## Conflict of Interest

The authors declare that the research was conducted in the absence of any commercial or financial relationships that could be construed as a potential conflict of interest.
