# Supplementary material for: Porphyromonas gingivalis Produce Neutrophil Specific Chemoattractants Including Short Chain Fatty Acids
Source: Front Cell Infect Microbiol. 2021 Jan 19;10:620681. doi: 10.3389/fcimb.2020.620681 (PMC7851090; doi:10.3389/fcimb.2020.620681)
Supplement: Supplementary file 2 [file DataSheet_2.pdf]

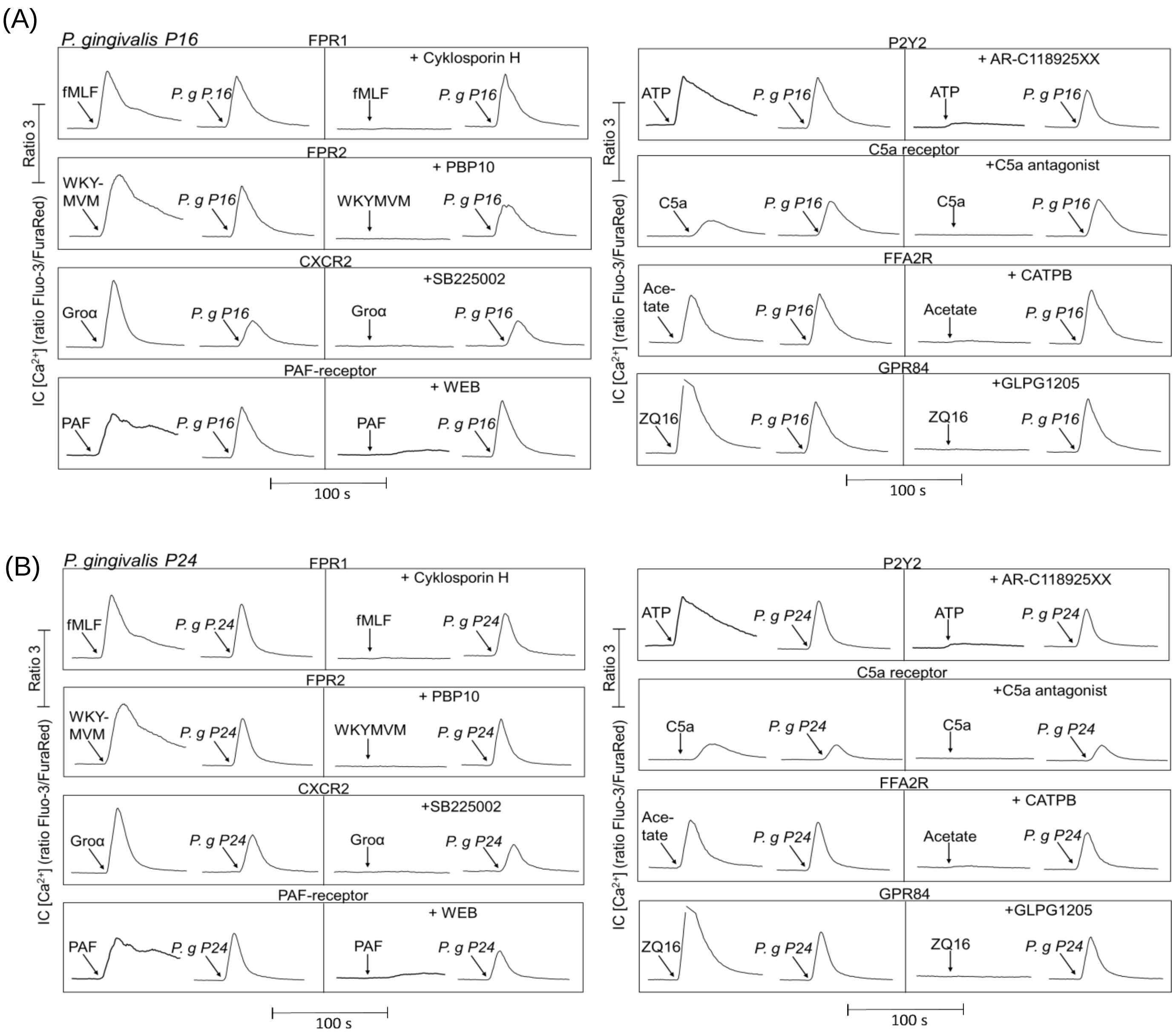

Supplementary figure 2. Intracellular Ca^{2+} signals induced by culture supernatants of *P. gingivalis* clinical isolates (P16 and P24) are not abrogated by inhibitors of well-known GPCRs. Human buffy-coat neutrophils were loaded with Fluo-3 and Fura-Red and the fluorescence ratio of the two dyes was monitored by flow cytometry. Cells were stimulated with culture supernatants of the two *P. gingivalis* clinical isolates (P16, P24) (1:20) and with GPCR-agonists after preincubation for 5 min at 37°C, untreated or treated with GPCR antagonists. **(A)** Results after stimulation with culture supernatant of *P. gingivalis* P16. **(B)** Results after stimulation with culture supernatant of *P. gingivalis* P24. The curves represent the fluorescence ratio between fluo-3 and Fura-Red and are representative of 3 independent experiments.
