# Supplementary material for: Porphyromonas gingivalis Produce Neutrophil Specific Chemoattractants Including Short Chain Fatty Acids
Source: Front Cell Infect Microbiol. 2021 Jan 19;10:620681. doi: 10.3389/fcimb.2020.620681 (PMC7851090; doi:10.3389/fcimb.2020.620681)
Supplement: Supplementary file 3 [file DataSheet_3.pdf]

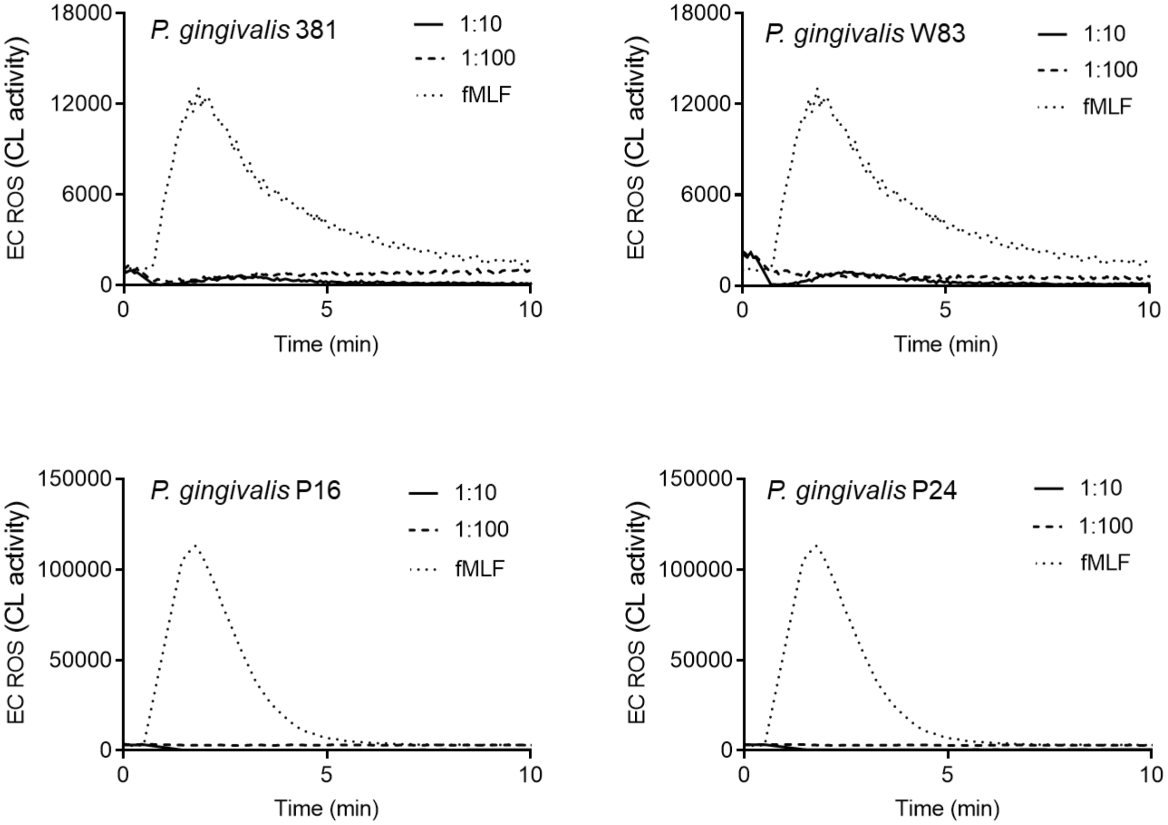


**Supplementary figure 3. Culture supernatants of *P. gingivalis* strains (381, W83, P16, P24) do not stimulate release of extracellular ROS in neutrophils.** Neutrophils separated from buffy-coats from healthy human blood donors were stimulated with culture supernatants of the four *P. gingivalis* strains (381, W83, P16, P24) (1:10, 1:100), fMLF (100 nM) or buffer. Extracellular superoxide production was detected with chemiluminescence amplified with isoluminol using a CLARIOstar plate reader (S/N 430-0226, BMG LABTECH). Stimulation with fMLF induced extracellular ROS production in neutrophils while no extracellular ROS production was triggered by the bacterial culture supernatants. The graphs are representative of 3 independent experiments .
