# Supplementary material for: Porphyromonas gingivalis Produce Neutrophil Specific Chemoattractants Including Short Chain Fatty Acids
Source: Front Cell Infect Microbiol. 2021 Jan 19;10:620681. doi: 10.3389/fcimb.2020.620681 (PMC7851090; doi:10.3389/fcimb.2020.620681)
Supplement: Supplementary file 4 [file DataSheet_4.pdf]

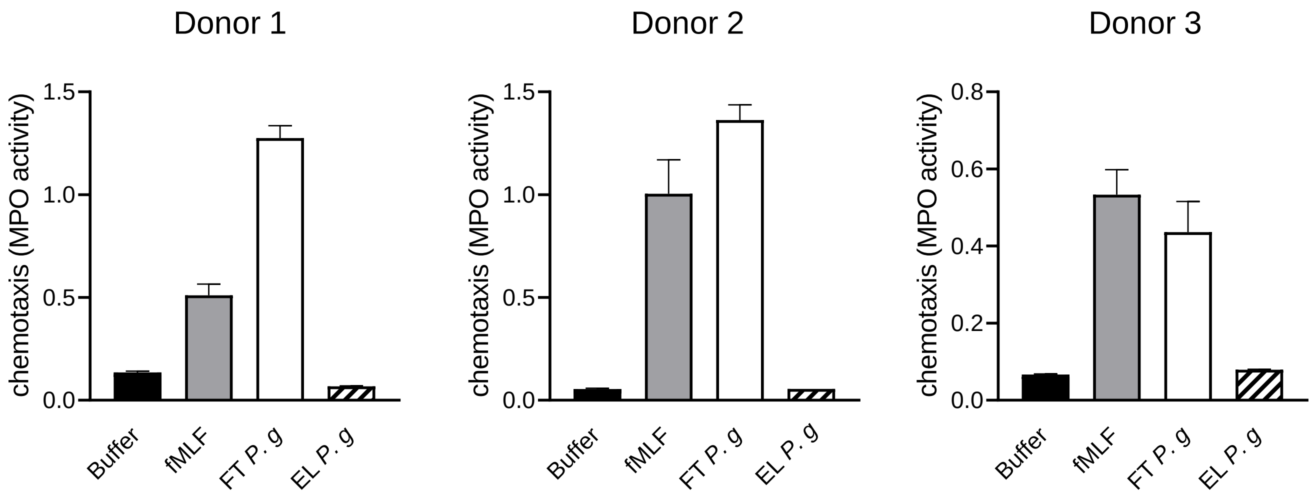

Supplementary figure 4. Neutrophil chemotaxis stimulated with the *P. gingivalis* EL and FT fractions. Peripheral blood neutrophils from healthy donors were allowed to migrate over a ChemoTx membrane stimulated with the FT and EL fractions of the hydrophobicity sorted *P. gingivalis* (381) supernatant, fMLF (10 nM) or buffer. The migrated cells were lysed and quantified by measurement of MPO activity. Graphs show neutrophil chemotaxis from 3 donors.
