# Supplementary material for: Porphyromonas gingivalis Produce Neutrophil Specific Chemoattractants Including Short Chain Fatty Acids
Source: Front Cell Infect Microbiol. 2021 Jan 19;10:620681. doi: 10.3389/fcimb.2020.620681 (PMC7851090; doi:10.3389/fcimb.2020.620681)
Supplement: Supplementary file 5 [file DataSheet_5.pdf]

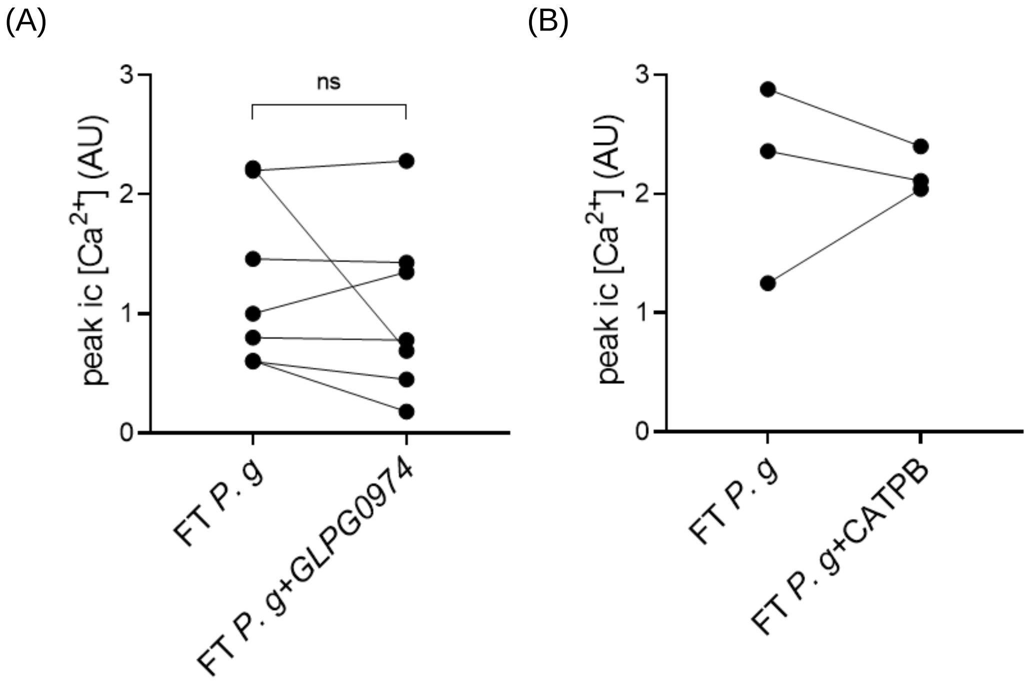

Supplementary figure 5. Effect of the FFA2R antagonists (GLPG0974 and CATPB) on intracellular calcium signals triggered by the *P. gingivalis* FT fraction. The FT fraction of the culture supernatant of *P. gingivalis* 381 was used to stimulate neutrophils in the presence or absence of a FFA2R antagonists (GLPG0974 or CATPB), and intracellular Ca^{2+} concentration was monitored by flow cytometry. Graphs show peak values of the neutrophil intracellular Ca^{2+} response after stimulation with the *P. g* FT fraction in presence or absence of GLPG0974 (n = 7) **(A)** or CATPB (n = 3) **(B).** Wilcoxon matched-pairs signed rank test was used to analyse statistics.
